# Nitrogen Supply Regulates Vascular Bundle Structure and Matter Transport Characteristics of Spring Maize Under High Plant Density

**DOI:** 10.3389/fpls.2020.602739

**Published:** 2021-01-08

**Authors:** Hong Ren, Ying Jiang, Ming Zhao, Hua Qi, Congfeng Li

**Affiliations:** ^1^Institute of Crop Science, Chinese Academy of Agricultural Sciences/Key Laboratory of Crop Physiology and Ecology, Ministry of Agriculture and Rural Affairs, Beijing, China; ^2^College of Agronomy, Shenyang Agricultural University, Shenyang, China

**Keywords:** spring maize, nitrogen supply, grain-filling, vascular bundle structure, matter transport efficiency

## Abstract

Nitrogen (N) fertilizer application greatly enhances grain yield by improving dry matter accumulation and grain filling in spring maize. However, how N application rates regulate the vascular bundle structure, matter transport and grain filling of spring maize under a high planting density has been poorly understood thus far. In this study, we analyzed the relationship between grain filling, vascular bundle structure and matter transport efficiency (MTE) of spring maize in the field. Zhongdan909 (ZD909) was used as the experimental material in a 2-year field experiment from 2015 to 2016, and it was grown under different N levels (0, 150, and 300 kg N ha^–1^) applied to the grain-filling stage of plots with planting densities of 67,500 plants ha^–1^ (ND) and 90,000 plants ha^–1^ (HD). Nitrogen application significantly optimized the structure of the big and small vascular bundles. In particular, there was an increase in the total number of small vascular bundles in the peduncle and cob of the ear system, i.e., increases of 51.8% and 25.7%, respectively, and the proportions of small vascular bundles to the total number of vascular bundles in the peduncle and cob were significantly increased. The root bleeding sap and MTE of maize were significantly increased by N application under both ND and HD, as indicated by the significant increase in the rate of ^13^C-photosynthate allocation to grain and amount of postsilking dry matter at maturity. Moreover, N application greatly improved the mean grain-filling rate (*G*_*mean*_) under ND and HD by 30.0% and 36.1%, respectively, and the grain-filling rate increased, leading to a distinct improvement in the grain sink at the grain-filling stage. We concluded that nitrogen application significantly optimized the vascular bundle structure of the ear system, increased the MTE and improved photosynthate distribution to the grain, ultimately enhancing the filling rate and grain yield.

## Introduction

Nitrogen (N) is the key nutrient element required for plant growth, and maize (*Zea mays* L.) production highly depends on the appropriate application of nitrogen fertilizer (Mu et al., 2016; Di Salvo et al., 2018). However, N requirement of maize is changing with various plant density. For instance, it is necessary to properly increase N input for enhancing dry matter accumulation and distribution and decreasing the competition of nutrients among individuals to increase the grain yield under high plant density, compared with normal plant density (Rajcan and Tollenaar, 1999; Wang et al., 2006; Ren et al., 2017). An insufficient N input will result in the issue of nutrient supply for plants, severely reduce the plant photosynthesis, accumulation of matter and grain yield (Tilman et al., 2002; Chen et al., 2011, 2015; Wei et al., 2017). Hence, the reasonable selection of N fertilizer input is beneficial to grain filling and grain weight in different periods after silking through coordinating crop photosynthesis, dry matter accumulation and distribution and others processes (Austin et al., 1997; Borrás et al., 2004). It has been recognized that the duration and rate at the reproductive growth stage determine the final grain weight and maize yield (Borrás et al., 2004; Zhou et al., 2017).

The grain-filling process can be divided into two stages: the first is the lag stage in which the kernel number and the potential kernel weight (size) are determined, and the second stage is the effective grain-filling stage in which the final kernel weight is established (Jones et al., 1996; Gambín et al., 2006; Hisse et al., 2019). The final kernel weight is highly dependent on the accumulation of assimilates during the initial grain-filling stages, but under stressful environmental conditions (such as low nitrogen and high planting density), the limitations of the supply of assimilates seriously influence the initial grain-filling stages and reduce the final kernel weight (Ouattar et al., 1987; Cirilo and Andrade, 1996; Gambín et al., 2006). Previous studies have focused on increasing the grain weight through nitrogen fertilizers by extending the effective filling period (Cao et al., 2008; Shi et al., 2013). However, Xu et al. (2016) pointed out that appropriate increasing nitrogen fertilizer can change the grain-filling rate and can increase the grain weight.

Actually, the N supply and planting density influence the potential assimilate supply and assimilate accumulation of maize grain, and matter transport efficiency (MTE) regulates the process of grain filling (Austin et al., 1997; Tollenaar et al., 2006; Wang et al., 2006). The assimilation of carbohydrates for grain-filled cereals are generally derived from two resources: (i) the current assimilates transferred directly to the grain and (ii) the assimilates redistributed from reserve pools in vegetative tissues either preanthesis or postanthesis (Gan and Amasino, 1997; Gebbing and Schnyder, 1999). However, under conditions associated with low nitrogen or high density, grain filling is limited by matter accumulation and increased transport (D’Andrea et al., 2016). As a result, the final grain weight decreases dramatically due to growth restrictions during the active grain-filling stage, and the accumulation of dry matter and the matter translocation rate have a decisive effect on grain filling (Ntanos and Koutroubas, 2002). Increasing studies have indicated that the main factor affecting maize grain filling under high-density conditions is not matter production but rather matter transport characteristics (Yang et al., 2002; Wang et al., 2019; Yan et al., 2019).

The smooth flow of matter transport depends on the structure and function of the vascular bundle systems above- and belowground (Zhong et al., 1997; Noguchi et al., 2005). It is generally accepted that xylem transports water and phloem transports organic matter (Shane et al., 2000). Organic matter, such as leaf photosynthetic products, is transported to the grain through the phloem, thereby achieving the purpose of increasing the grain weight, and the phloem unloads 60–98.2% of total carbon into the grain (Khan et al., 1998; Wigoda et al., 2014). Higher numbers and areas of vascular bundles can regulate the distribution of dry matter after silking by regulating the metabolism of carbon (C) and N and, to a certain extent, can determine the accumulation of C and N in the grain and the size of the grain sink, which affects grain filling under various conditions (Housley and Peterson, 1982; Yang et al., 2001; Monneveux et al., 2005). A previous study showed that tillage practice and row spacing could optimize the structure of the vascular bundles, improve matter transport and thus increase maize yield (Piao et al., 2016). However, the mechanism of how N application under high-density conditions regulates the grain-filling efficiency of maize by affecting vascular bundle characteristics is unclear.

Therefore, the purpose of this study is to clarify three points: (1) how the N and plant density interaction influences grain filling and matter transport characteristics, (2) how the N and plant density interaction affects the vascular bundle structure, and (3) the relationship between grain filling and vascular bundle structure under various N fertilizer conditions.

## Materials and Methods

### Site Description

The field experiment was conducted in Gongzhuling, Jilin Province, China (43°31′N, 124°48′E) in 2015 and 2016. The experimental soil (brown loam) consisted of organic matter 25.9 g kg^–1^, total N 1.6 g kg^–1^, available N 140.2 mg kg^–1^, available P 62.3 mg kg^–1^, and available K 148.40 mg kg^–1^. Precipitation (mm) and mean air temperatures measured during the growth stage of spring maize are shown in Supplementary Figure 1. The effective cumulative temperature was calculated using the mean daily air temperature above 10°C; the respective rainfall and effective cumulative temperature during the growth stage of spring maize were 409.6 mm and 1631.3°C in 2015 and 643.7 mm and 1616.0°C in 2016.

### Experimental Design and Field Management

The field experiment with a split plot design was performed with two planting densities (67,500 plants ha^–1^, ND; 90,000 plants ha^–1^, HD) as the main plot and three N levels, 0 kg N ha^–1^ (N0), 150 kg N ha^–1^ (N150), and 300 N kg ha^–1^ (N300), as subplots. All treatments were set with three replicates. The size of each plot was 6 m × 7.5 m with 0.6 m row spacing. The application of N fertilizer (urea) was performed before sowing at the elongation and silking stages of maize at a 5:3:2 ratio. Both Ca_3_(PO_4_)_2_ and KCl were applied before sowing at 100 kg ha^–1^ in 2015 and 2016. Pests, weeds and diseases were well controlled, and no irrigation was applied throughout the growing season. Maize seed was planted with hand planters on 30 April 2015 and 30 April 2016, and the respective harvest dates were October 1, 2015 and 2016.

### Data Collection

#### Characterization of Vascular Bundles and Matter Transport Efficiency

The characterization of the vascular bundles of maize was performed according to the description provided by He et al. (2005). Briefly, at the milking stage, five random plants in each plot were manually sampled and dissected to obtain the basal stem (1 cm above the ground), ear internodes, peduncle and cob fractions. All samples collected were fixed using Kano fixative solution containing acetic acid/alcohol = 1:3 (v/v) and then stored in 70% ethanol until imaging. Freehand sectioning, safflower staining, and observation of the vascular bundle structure were performed with a Zeiss Axio A1 upright microscope with a 5×/0.3 numerical aperture (NA) and 10×/0.3 NA Axio HRc camera (Carl Zeiss Inc., Ontario, CA, United States). The numbers of small vascular bundles (average of 18 adjacent vascular bundles) were recorded, and the built-in ZEN analysis system was used to calculate the area occupied by the vascular bundles in both the xylem and phloem.

To determine the MTE, root-bleeding sap was sampled from the above maize plants by using lopping shears to cut 1 cm above the basal stem internode of five representatives per plot. Twelve grams of absorbent cotton was wrapped around the basal stem and secured with rubber bands to collect the root-bleeding sap. The weight of the root-bleeding sap was measured after 12 h of collection (from 17:00 to 05:00). Then, the MTE (mg mm^–2^h^–1^) was calculated as follows, Eq. 1 (Piao et al., 2016).

(1) M⁢T⁢E=R⁢B⁢S/V⁢B⁢A

where RBS refers to the root-bleeding sap collected from 17:00 to 05:00 of the next day (mg h^–1^) and VBA refers to the vascular bundle area of the basal stem internode (mm^2^).

#### Grain-Filling Parameters

During the grain-filling stage, three plants per plot were randomly sampled. In 2015, the N0 treatment was sampled at 10, 25, 32, 43, and 57 days after pollination for the ND and HD plots; the N150 and N300 treatments were sampled at 10, 20, 30, 38, and 53 days after pollination for the ND plot and at 10, 20, 30, 40, and 55 days after pollination for the HD plot. In 2016, the N0 treatment was sampled at 7, 14, 24, 33, 48, and 56 days after pollination for the ND plot and at 7, 14, 24, 31, 46 and 56 days after pollination for the HD plot; and the N150 and N300 treatments were sampled at 7, 14, 25, 32, 47, and 56 days after pollination for the ND and HD plots. We sampled 100 kernels in the middle of the ear according to the grain position; these kernels were dried to a constant weight at 80°C. The dry matter accumulation of the kernel was estimated by fitting a logistic equation (Eq. 2) to the dry kernel weight plotted against the number of days after pollination during the grain-filling stage (Cao et al., 2008).

(2) y=a/(1+b×exp⁢(-c⁢x))

where *x* is the number of days after pollination, *y* is the dry weight of one kernel (g), and *a*, *b*, and *c* are estimated parameters representing the ultimate growth mass, primary parameter, and growth rate parameter, respectively. The following equation (Eq. 3), derived by taking the first derivative of Eq. 2, was used to estimate the effective grain-filling duration and kernel growth rate:

(3) $$d\,y/d\,x=a\times b\times c\times \text{exp}\,\left(-\text{cx}\right)/{\left(1+b\times \exp\left(-\text{cx}\right)\right)}^{2}$$

The estimated parameters *a*, *b*, and *c* were used to calculate the grain-filling parameters. The day reaching the rate maximum grain-filling rate is *T*_*max*_ = *ln*⁡*b*/*c*, the increase in the weight of the kernel at the maximum grain-filling rate is *W*_*max*_=*a*/2, the maximum grain-filling rate is *G*_*max*_ =a×c/2, the mean grain-filling rate is *G*_*mean*_ =c×*W*_*max*_/6, the active grain-filling phase is *P* =  6/c, and the lag grain-filling period is *T*_1_ = (*ln*⁡*b*−1.317)/*c*.

#### ^13^C-Photosynthate Allocation Measurements Using ^13^C Isotope Labeling

To determine the ^13^C-photosynthate distribution among plant organs, 10 individual plants in each plot were selected for ^13^C labeling at the silking (R1) stage of maize. Carbon dioxide-^13^C (SRICI, Shanghai, China) with an enrichment >99 atom% was used for the tracing test. Ear leaves of selected plants were encased within 0.1-mm-thick mylar plastic bags, which allowed sunlight transmission at levels up to 95% of natural intensity. The bags were sealed with plastic tape at the base and then injected with 50 ml ^13^CO_2_. The bags were removed after plant photosynthesis proceeded for 1 h. After 24 h, five labeled plants were sampled at ground level, and the remaining plants were collected at maturity (R6) stage. All labeled plants were dissected into stems, sheaths, ear leaves, other leaves, cobs and grains, oven-dried at 80°C to a constant weight, weighed to determine the dry matter, and then finely ground into powder. Subsequently, 5 mg of powder was used to determine the ^13^C isotopic abundance using an Isoprime 100 instrument (Isoprime 100, Cheadle, United Kingdom). The calculated ^13^C atom% excess in the plant organs refers to the distribution of ^13^C in the organs after ^13^CO_2_ feeding, as described in previous studies (Wei et al., 2009; Liu et al., 2015).

The natural abundance of ^13^C% was calculated as follows (Eq. 4).

(4) $$\delta^{13}\,C\%\,=\,\left(R_{s\,a\,m\,p\,l\,e}/R_{P\,B\,D}-1\right)\times 100$$

where *R*_*sample*_ = ^13^*C*/^12^*C*_*sample*_ and *R*_*PDB*_ = ^13^*C*/^12^*C*_*PDB*_, which has a value of 0.0112372. The labeled plant ^13^C abundance was calculated as follows (Eq. 5):

C13(%)=(δ13C+100)×RP⁢B⁢D/

(5) ×100

According to Eqs. 4 and 5, we calculated the ^13^C mg excess in each organ as follows (Eq. 6):

C13⁢m⁢g⁢e⁢x⁢c⁢e⁢s⁢s

=(dryweightofplanteachorgan(mg)×C%)×

(6) (C%13-δ13⁢C%)×100

The distribution of the ^13^C excess ratio in each plant organ 24 h after ^13^CO_2_ feeding and at maturity (R6) was calculated as follows (Eq. 7):

C13(%)=13Cmgexcessineachplantorgan/

(7) $$t\,o\,t\,a\,l^{13}\,C\,m\,g\,e\,x\,c\,e\,s\,s\,i\,n\,t\,h\,e\,p\,l\,a\,n\,t\times 100$$

#### Dry Matter Accumulation and Grain Yield Measurement

In each plot, five individual plants were cut manually at ground level to determine the dry matter (DM) aboveground at the silking and maturity stages in 2015 and 2016. The harvested plants were dried initially at 105°C for 1 h to deactivate enzymes and then oven-dried at 80°C to a constant weight before weighing. The DM translocation efficiency of vegetative organs (%) (DMTE) was calculated for the postsilking stage following previous studies (Zhu et al., 2011) (Eq. 8).

DMTE(%)= 100×(DMallvegetativeorgansatsilking

-DMofallvegetativeorgansatmilkingormaturitystage)/

(8)  ⁢D⁢M⁢a⁢l⁢l⁢v⁢e⁢g⁢e⁢t⁢a⁢t⁢i⁢v⁢e⁢o⁢r⁢g⁢a⁢n⁢s⁢s⁢i⁢l⁢k⁢i⁢n⁢g

At physiological maturity, maize was harvested manually in a bordered area (5 m × 3.6 m) of each plot to determine grain yield. The grain yield was adjusted to 14% moisture content. The kernel number per ear was recorded, and five samples were randomly selected from each plot and then air-dried to record the 1000-kernel weight.

### Statistical Analysis

Curve Expert 1.3 software was used to obtain the grain-filling logistic equation and parameters *a*, *b*, and *c*. Analysis of variance (ANOVA) was performed using SPSS 20.0 software (SPSS Institute Inc., United States), and figures were produced by Origin 9.0 and R 3.4.1.

## Results

### Characteristics of the Vascular Bundle in Internodes

**FIGURE 1:**
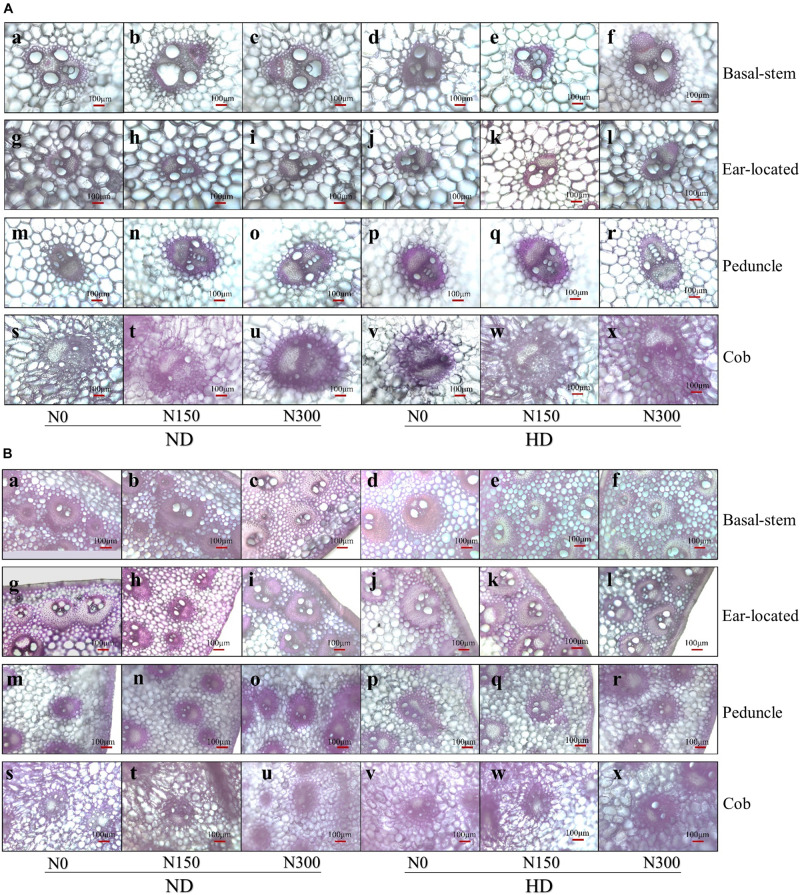
**(A)** The micrograph of big vascular bundles structure at basal stem internode **(a–f)**, ear located **(g–i)**, peduncle **(m–r)**, and cob **(s–x)** internodes. ND and HD indicate planting density of 67,500 and 90,000 plants ha^− 1^; N0, N150, and N300 indicate N applied at 0, 150, and 300 kg ha^− 1^ levels, respectively. **(B)** The micrograph of small vascular bundles structure at basal-stem internode **(a–f)**, ear located **(g–i)**, peduncle **(m–r)**, and cob **(s–x)** internodes. ND and HD indicate planting density of 67,500 and 90,000 plants ha^− 1^; N0, N150, and N300 indicate N applied at 0, 150, and 300 kg ha^− 1^ levels, respectively.

**FIGURE 2:**
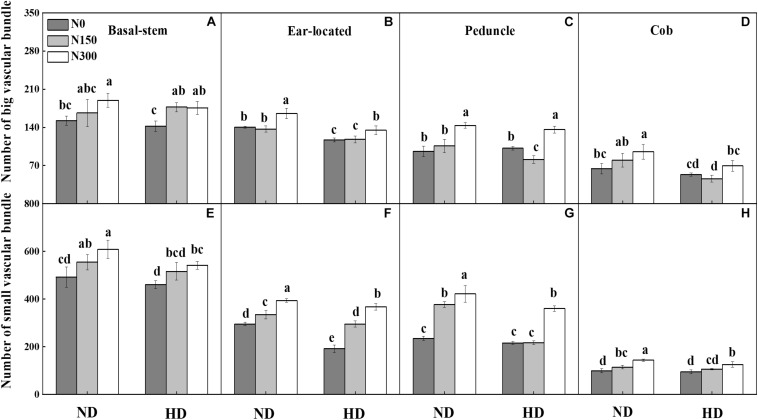
Effect of planting density and nitrogen application on number of big **(A–D)** and small **(E–H)** vascular bundles at maize R3 stage in 2016. ND and HD indicate planting density of 67,500 and 90,000 plants ha^− 1^; N0, N150, and N300 indicate N applied at 0 kg N ha^− 1^, 150 kg N ha^− 1^, and 300 kg N ha^− 1^ levels, respectively. Different letters indicate significant difference at 5% level. Values shown are the mean ± SE (*n* = 3).

The microscopic structure of vascular bundles from the basal stem, ear-located, peduncle and cob internodes of maize in 2016 is shown in Figures 1A,B. Generally, the numbers of both big and small vascular bundles gradually decreased from the basal stem to cob fractions (Figure 2). Planting density tended to decrease the numbers of both big and small vascular bundles in all internode fractions of maize. For instance, the numbers of big vascular bundles in cobs was decreased by an average of 28.8%, and small vascular bundles in peduncles was decreased by an average of 21.8% (Figures 2D,G). Compared with N0, increased N input significantly increased the number of vascular bundles, by an average of 20.7%, 7.9%, 18.2%, and 21.9% for big vascular bundles and 16.5%, 48.1%, 51.8%, and 25.7% for small vascular bundles in the basal stem, ear-located, peduncle and cob, respectively (*P* < 0.05).

Planting density and N fertilization level significantly influenced vascular bundles in the phloem and xylem, including the total area of the vascular bundles (TAVB) and the proportions of big vascular bundles (PBVB) and small vascular bundles (PSVB) relative to the total vascular bundle area (Table 1, *P*<0.05). The average values of PBVB in both the basal stem (0.36) and ear location (0.45) were lower than those of PSVB (0.64 and 0.55). However, the single average area of small vascular bundles decreased and the single average area of big vascular bundles increased at the cob internode, resulting in PBVB in both the peduncle (0.45) and cob (0.30) being higher than those of PSVB (0.55 and 0.70). Through increasing the area of the small vascular bundles, the PSVB was enhanced by N application increased, especially in the peduncle and cob internodes. In the peduncle and cob, the average single area of small vascular bundles increased by 53.4% and 102.6% while the average single area of vascular bundles increased by only 16.2% and 63.6%, respectively. Moreover, the average single area of small vascular bundles in the phloem increased by 34.8% in the peduncle and 35.0% in the cob while this area in the xylem increased by 27.4% in the peduncle and 18.4% in the cob.

**TABLE 1:** Effect of planting density and nitrogen application on vascular bundle area of maize at milking stage in 2016.

**Position**	**Planting density**	**N level**	**The average area of single big vascular bundle (×10^–3^ mm^2^)**			**The average area of single small vascular bundle (×10^–3^ mm^2^)**			**TAVB**	**PBVB**	**PSVB**
							
			**Sum**	**Phloem**	**Xylem**	**Sum**	**Phloem**	**Xylem**	**(mm^2^)**		
Basal-stem	ND	N0	63.1d	7.3c	26.4c	48.8c	2.4b	11.7b	33.7d	0.30c	0.70a
		N150	89.9bc	9.3b	32.3b	62.9a	2.5b	10.3b	49.8b	0.30c	0.70a
		N300	122.6a	13.5a	49.8a	63.8a	3.4a	15.6a	61.9a	0.37b	0.63b
	HD	N0	79.0cd	5.4c	31.0b	47.2c	2.3b	9.7b	33.0d	0.34b	0.66b
		N150	97.3b	6.0c	29.8bc	48.2c	2.6bc	9.4b	41.1c	0.42a	0.58c
		N300	135.4a	13.1a	51.0a	53.0b	2.3b	14.4a	53.5b	0.44a	0.56c
Ear-located	ND	N0	59.8c	5.3c	17.4c	40.4b	1.7b	6.9a	20.3d	0.41bc	0.59ab
		N150	77.1b	5.9bc	17.0c	40.8b	2.2a	7.1a	24.2c	0.44bc	0.56ab
		N300	89.0a	8.2a	20.9a	46.3a	2.2a	7.5a	33.0a	0.45b	0.55b
	HD	N0	76.9b	6.1bc	13.2d	37.1c	0.5c	5.3b	16.1e	0.56a	0.44c
		N150	83.0ab	6.5b	19.8ab	42.6b	2.0ab	6.8a	22.4cd	0.44bc	0.56ab
		N300	85.3a	6.9b	18.1bc	46.2a	2.1ab	7.0a	28.5b	0.40c	0.60a
Peduncle	ND	N0	83.3cd	13.7d	23.3d	24.1c	2.2c	4.6c	13.7d	0.58b	0.42c
		N150	77.3d	15.2cd	26.5c	24.2c	2.6bc	4.7c	17.3c	0.49c	0.51b
		N300	109.3a	18.6b	30.0b	45.9a	3.5a	6.8a	32.8a	0.45d	0.55a
	HD	N0	85.7bc	18.2b	22.6d	19.2d	2.1c	4.2c	12.9de	0.68a	0.32d
		N150	92.6b	17.0bc	28.9bc	22.3c	2.4c	4.8c	12.3e	0.59b	0.41c
		N300	113.7a	21.4a	41.7a	39.7b	3.1ab	6.1b	29.8b	0.52c	0.48b
Cob	ND	N0	82.8d	12.7c	29.8b	22.3e	2.9c	6.3c	7.6c	0.73a	0.27c
		N150	152.7b	17.3b	41.6a	47.3b	3.5bc	6.8bc	17b	0.69b	0.31b
		N300	169.6a	19.6b	45.1a	58.6a	4.6ab	8.1a	24.5a	0.65c	0.35a
	HD	N0	121.2c	19.1b	22.7c	21.7e	2.8c	6.2c	8.5c	0.76a	0.24c
		N150	144.5b	18.1b	44.7a	28.1d	3.3bc	7.5ab	9.6c	0.69b	0.31b
		N300	176.7a	23.6a	43.0a	44.7c	4.0b	7.2b	17.9b	0.66bc	0.34ab

*TAVB, total area of vascular bundle; PBVB, proportion of big vascular bundle area to total vascular bundle area; PSVB, proportion of small vascular bundle area to total vascular bundle area. ND and HD indicate planting density of 67,500 and 90,000 plants ha^–1^; N0, N150, and N300 indicate N applied at 0, 150, and 300 kg ha^–1^ levels, respectively. Values of six treatments followed by different letters indicate significant difference at 5% level.*

### Root-Bleeding Sap and Matter Transport Efficiency

**FIGURE 3:**
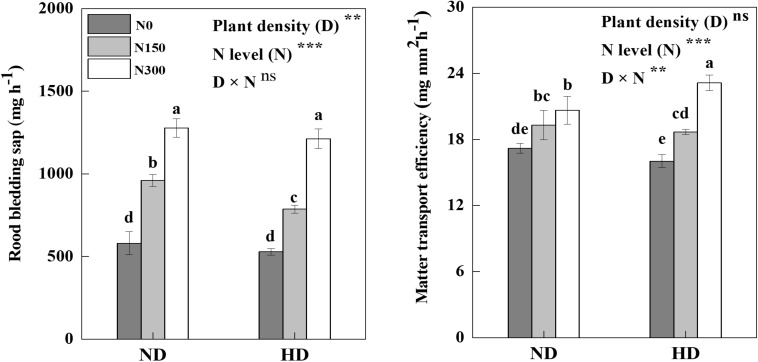
Response of root bleeding sap and matter transport efficiency on planting density and nitrogen fertilization level at maize R3 stage in 2016. ND and HD indicate planting density of 67,500 and 90,000 plants ha^− 1^; N0, N150, and N300 indicate N applied at 0, 150, and 300 kg ha^− 1^ levels, respectively. Different letters indicate significant difference at 5% level. Values shown are the mean ± SE (*n* = 3). ns, no significance; *, **, and *** indicate significance at 5%, 1%, and 0.1% level, respectively.

Root-bleeding sap was significantly affected by planting density (*D*) and N fertilizer level (*N*), and MTE was significantly affected by the N application level and *D* × *N* (Figure 3, *P* < 0.05). Compared with N0, root-bleeding sap and MTE under both ND and HD conditions were significantly increased by N application, the averages increase of 93.0% and 89.3% for root-bleeding sap and 16.3% and 30.4% for MTE, respectively (*P* < 0.05). In particular, the highest MTE value (23.1 mg mm^–2^ h^–1^) was measured under HD in the N300 treatment. Compared with ND treatment, HD increased MTE by 11.6% under N300 conditions.

### Grain-Filling Characteristics

The factors year (*Y*), planting density (*D*) and N level (*N*) significantly affected the grain-filling parameters. including the kernel weight increment achieving maximum grain-filling rate (*W*_*max*_), the day reaching the maximum grain-filling rate (*T*_*max*_), the maximum filling rate (*G*_*max*_), the active grain filling phase (*P*), the mean grain-filling rate (*G*_*mean*_), and the lag period (T1) (Table 2). In addition, Tmax and T1 were significantly affected by *D* × *N*, and except for *G*_*max*_, other grain-filling parameters were significantly affected by *Y* × *D* × *N* (*P* < 0.05). The *G*_*max*_, *P*, and *G*_*mean*_ were significantly improved by N application increased, especially under HD conditions. Compared with N0, the *G*_*max*_, *P*, and *G*_*mean*_ under HD conditions were average 35.2%, 8.1%, and 36.1% increased by N150 and N300 treatments, and these values were observed with 29.8%, 10.5%, and 30.0% increased under ND condition. Comparing those grain-filling parameters between ND and HD, the *G*_*max*_, *P*, and *G*_*mean*_ under HD condition were average 8.1%, 1.8%, and 7.8% lower than those under ND condition in 2015 and 2016. Moreover, the effects of the effects of planting density and N input on the on the *G*_*mean*_ were more obvious.

**TABLE 2:** Effect of planting density and nitrogen application on grain filling parameters of maize in 2015 and 2016.

**Year**	**Planting density**	**N level**	***W*_*max*_**	***T*_*max*_**	***G*_*max*_**	***P***	***G*_*mean*_**	***T*_1_**
			**(g 100-kernel^–1^)**	**(d)**	**(g d^–1^ 100-kernel^–1^)**	**(d)**	**(g d^–1^ 100-kernel^–1^)**	**(d)**
2015	ND	N0	11.87d	28.02c	1.76d	40.41c	0.29d	19.15b
		N150	15.77b	26.13d	2.14b	44.24b	0.36b	16.42d
		N300	17.93a	26.20d	2.36a	45.55b	0.39a	16.20d
	HD	N0	10.71e	33.53a	1.61e	40.04c	0.27e	24.74a
		N150	14.71c	28.93b	2.00c	44.25b	0.33c	19.21b
		N300	17.66a	28.25bc	2.21b	47.97a	0.37b	17.71c
2016	ND	N0	11.37d	28.53a	1.86e	36.62c	0.31e	20.49a
		N150	14.37c	22.18d	2.29c	37.62bc	0.38c	13.93bc
		N300	18.28a	22.98bc	2.61a	42.82a	0.43a	13.75c
	HD	N0	10.88d	28.81a	1.65f	39.60bc	0.27f	20.12a
		N150	13.90c	23.07bc	2.15d	38.79bc	0.36d	14.56b
		N300	16.91b	23.35b	2.46b	41.29ab	0.41b	14.31bc
ANOVA								
Year (*Y*)			***	***	***	***	***	***
Plant density (*D*)			***	***	***	***	***	***
N level (*N*)			***	***	***	*	***	***
*D × N*			ns	***	ns	ns	ns	***
*Y* × *D* × *N*			*	***	ns	***	*	***

W_*max*_, the kernel weight increment achieving maximum grain-filling rate; *T*_*max*_, the day reaching the maximum grain-filling rate; *G*_*max*_, the maximum filling rate; *P*, the active grain filling phase; *G*_*mean*_, the mean grain-filling rate; *T*1, the lag period. ND and HD indicate planting density of 67,500 and 90,000 plants ha^–1^; N0, N150, and N300 indicate N applied at 0, 150, and 300 kg ha^–1^ levels, respectively. Values of six treatments followed by different letters indicate significant difference at 5% level. ns, no significance; *, **, and *** indicate significance at 5%, 1%, and 0.1% level, respectively.

**FIGURE 4:**
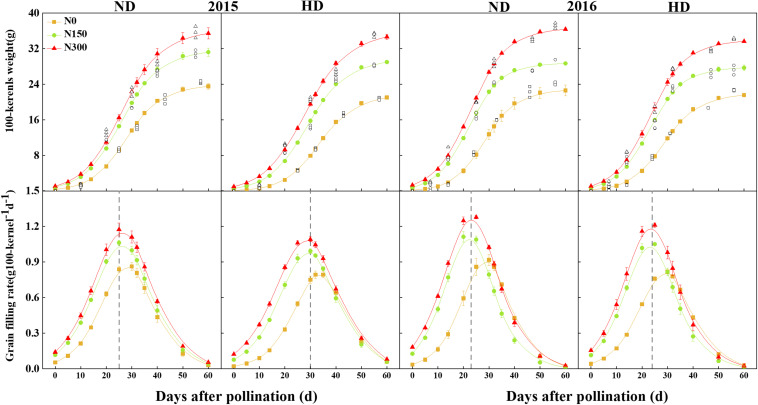
Changes of grain filling rate and 100-kernel weight of maize affected by planting density and nitrogen fertilization level in 2015 and 2016. The reference line represents the number of days to reach the peak of grain filling. ND and HD indicate planting density of 67,500 and 90,000 plants ha^− 1^; N0, N150, and N300 indicate N applied at 0, 150, and 300 kg ha^− 1^ levels, respectively. Values shown are the mean ± SE (*n* = 3).

According to the grain-filling parameters, the grain-filling process of maize was well simulated using logistic equations. In Figure 4, N input significantly increased the kernel weight (sink): in the initial grain-filling stages (the sink size was defined), the kernel weight increased by 42.7–46.3%, and the completion time of the lag period was 3–7 days earlier than that of the N0 treatment. HD resulted in a 1- to 5-day delay when the peak grain-filling rate was reached compared with ND.

### ^13^C-Photosynthate Allocation Between Maize Organs at Silking and Maturity

**FIGURE 5:**
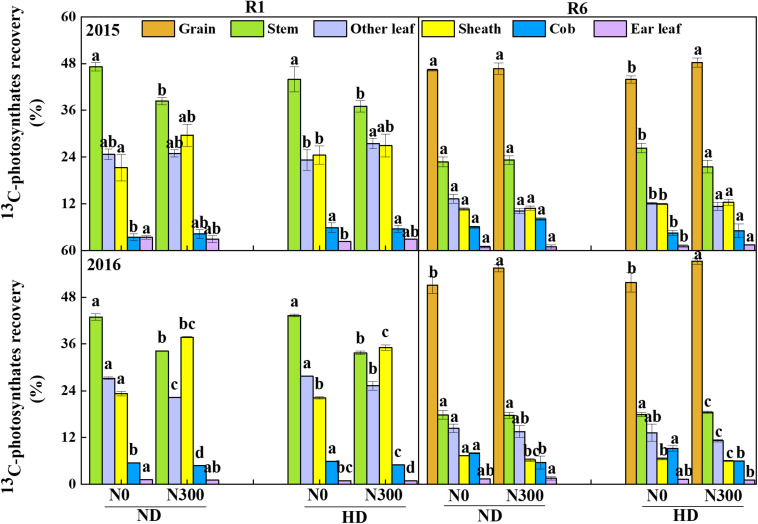
^13^C-photosynthates allocation between maize organs at R1 and R6 stages in 2015 and 2016. ND and HD indicate planting density of 67,500 and 90,000 plants ha^− 1^; N0, N150, and N300 indicate N applied at 0, 150, and 300 kg ha^− 1^ levels, respectively. Different letters indicate significant difference at 5% level. Values shown are the mean ± SE (*n* = 3).

Generally, after ^13^C labeling for 24 h, the highest ratio of ^13^C-photosynthates in maize shoot at silking stage was recorded in stem, followed by other leaves and sheath, cob and ear leaves were found with very low ^13^C-photosynthates distribution (Figure 5). It was interesting to find that higher ^13^C-photosynthates distribution of N0 treatment in stem than those of N300 treatment under both ND and HD conditions. At maturity stage, the highest ratio of ^13^C-photosynthates allocation to grain from N300 treatment under HD condition were 48.3% and 57.3% in 2015 and 2016. Compared with N0, the ^13^C-photosynthates allocations to grain from N300 treatment were increased by 0.7–10.5% in 2015 and 2016.

### Yield Components and Dry Matter Accumulation

According to ANOVA results, the factors year (*Y*), planting density (*D*), N level (*N*) and their interactions (*D* × *N* and *Y* × *D* × *N*) had significant effects on the 1000-kernel weight (TKW) and grain yield (Table 3). N fertilizer input significantly increased the kernel number per ear (KNP) and TKW under both ND and HD (*P* < 0.05), by 23.7% and 38.8% for KNP and by 17.1% and 12.8% for TKW in 2015 and 2016, respectively. The highest grain yields were 11.0 Mg ha^–1^ and 12.3 Mg ha^–1^, obtained from the N300 treatment under HD conditions in 2015 and 2016, respectively.

**TABLE 3:** Effect of planting density and nitrogen application on yield components of maize in 2015 and 2016.

**Year**	**Planting density**	**N level**	**Kernel number**	**1000-Kernel weight**	**Grain yield**
			**(ear^–1^)**	**(g)**	**(Mg ha^–1^)**
2015	ND	N0	457.0d	285.9e	5.2f
		N150	535.7b	316.0c	7.4d
		N300	573.0a	351.2a	9.2b
	HD	N0	344.2f	279.9e	6.5e
		N150	435.2e	305.7d	8.7c
		N300	459.3c	334.4b	11.0a
2016	ND	N0	424.0c	293.9d	5.1f
		N150	529.7a	318.3c	9.4d
		N300	540.4a	372.4a	11.9b
	HD	N0	342.0d	292.3d	7.0e
		N150	503.7b	317.1c	10.8c
		N300	506.9b	333.1b	12.3a
**ANOVA**					
Year (*Y*)			ns	***	***
Plant density (*D*)			***	***	***
N level (*N*)			***	***	***
*D* × *N*			ns	***	***
*Y* × *D* × *N*			***	***	***

ND and HD indicate planting density of 67,500 and 90,000 plants ha^–1^; N0, N150, and N300 indicate N applied at 0, 150, and 300 kg ha^–1^ levels, respectively. Values of six treatments followed by different letters indicate significant difference at 5% level. ns, no significance; *, **, and *** indicate significance at 5%, 1%, and 0.1% level, respectively.

**FIGURE 6:**
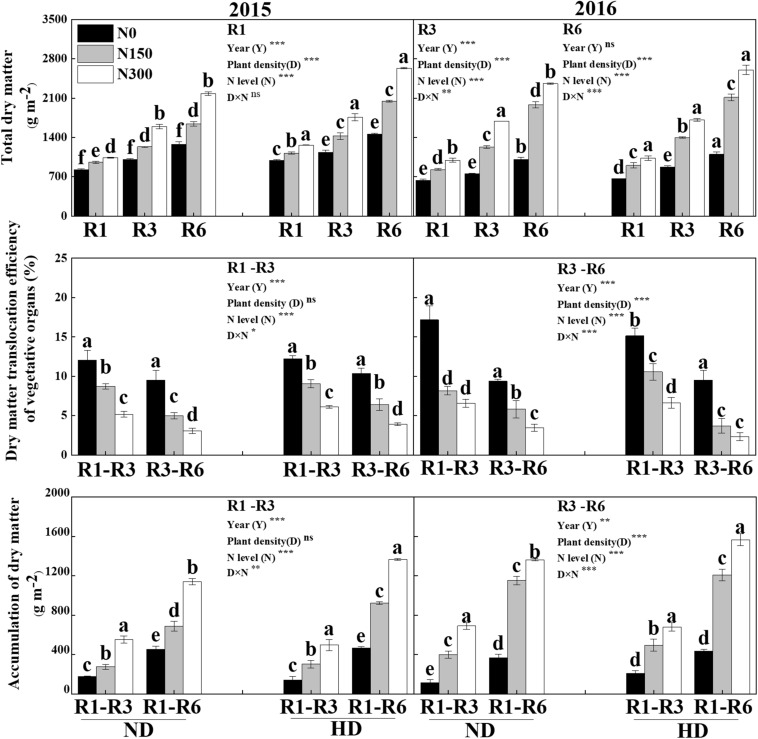
Effect of planting density and nitrogen application on the accumulation and translocation of dry matter during maize R1, R3, and R6 stages in 2015 and 2016. ND and HD indicate planting density of 67,500 and 90,000 plants ha^− 1^; N0, N150, and N300 indicate N applied at 0 kg N ha^− 1^, 150 kg N ha^− 1^, and 300 kg N ha^− 1^ levels, respectively. Different letters indicate significant difference at 5% level. Values shown are the mean ± SE (*n* = 3); ns, no significance; *, **, and *** indicate significance at 5%, 1%, and 0.1% level, respectively.

Significant effects on total dry matter (DM) were obtained from both planting density and N level at the silking (R1), milking (R3) and maturity (R6) stages of maize, and the accumulation of DM and its translocation were significantly different under the different levels of N and its interaction with the planting density treatments. Total DM was significantly increased with increasing N application (*P* < 0.05), especially under HD, by 33.0% at R1, 59.0% at R3 and 87.1% at R6. Similarly, the Postsilking DM was significantly increased by N input, compared with N0, the postsilking DM, i.e., an increase of 218.1% in the accumulation of DM from R1 to R3 and of 161.4% from R3 to R6, and of the 170.3% from R1 to R6. However, N input decreased the dry matter translocation efficiency of vegetative organs (DMTE) from R1 to R3 and from R3 to R6 by 45.1% and 56.7%, respectively (Figure 6).

### Correlation Analysis

**FIGURE 7:**
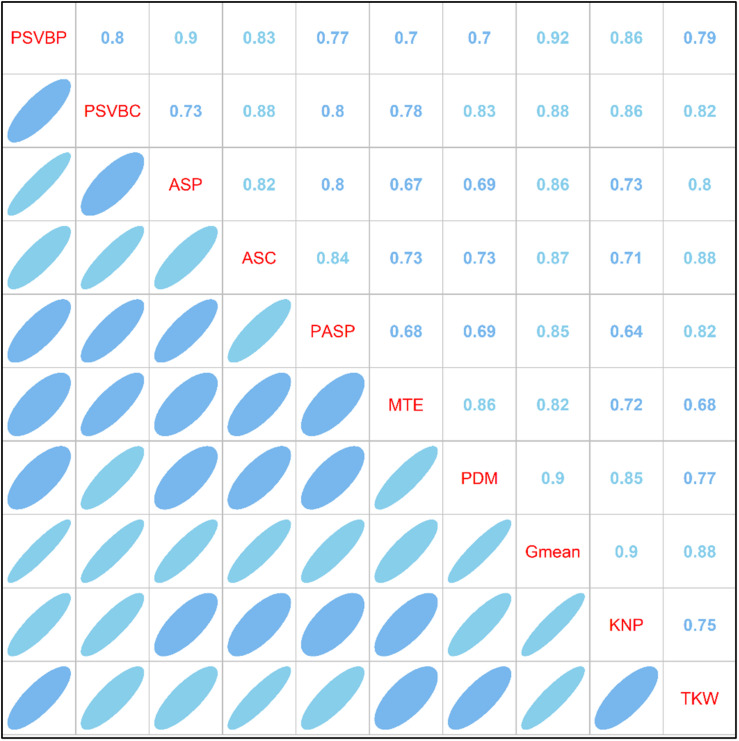
Correlation analysis of post-silking dry matter, yield components, vascular bundle structure and grain filling parameters under different planting density and nitrogen fertilization levels in 2016. PBVBP and PSVBC, the proportion of small vascular bundle area to total vascular bundle area in peduncle and cob; ASP and ASC, the number of small vascular bundle in peduncle and cob; PASP, the phloem area of small vascular bundle in peduncle; MTE, matter transport efficiency of basal-stem internode; *G*_*mean*_, mean grain-filling rate; PDM, the accumulation of postsilking dry matter (R1–R6); TKW, 1000-kernel weight; KNP, kernel number per ear.

As shown in Figure 7, correlation analysis between the vascular bundle structure, MTE, the accumulation of postsilking dry matter (PDM) and mean grain-filling rate (*G*_*mean*_) revealed that MTE was positively correlated with PDM and *G*_*mean*_ (*R*_*MD*_ = 0.86, *R*_*MG*_ = 0.82), and both PDM and *G*_*mean*_ were positively correlated with KNP and TKW (*R*_*DKN*_ = 0.85, *R*_*DKW*_ = 0.77; *R*_*GKN*_ = 0.90, *R*_*GKW*_ = 0.88). Correlation analysis showed that the proportion of the small vascular bundle area to the total vascular bundle area in the peduncle (PSVBP) and cob (PSVBC) and the corresponding number of vascular bundles in the peduncle (ASP) and cob (ASC) were positively correlated with MTE (*R*_*PM*_ = 0.70, *R*_*CM*_ = 0.78; *R*_*APM*_ = 0.67; *R*_*ACM*_ = 0.73). Additionally, in the phloem of the peduncle, the area of small vascular bundles (PASP) was positively correlated with TKW (*R*_*SM*_ = 0.82).

## Discussion

Grain filling is an important process in determining grain yield that can explain yield changes, which influenced by agricultural management, such as N input and planting density (Xu et al., 2013; Wei et al., 2017, 2019). An appropriate increase in N application could improve the photosynthate supply and matter transport from vegetative to reproductive organs, contributing to the grain-filling rate, grain-filling phase and grain yield under high-density planting conditions (He et al., 2005; Ciampitti and Vyn, 2012; Hou et al., 2012). Grain filling is determined by the grain-filling rate and the active grain-filling phase (Zhou et al., 2017). Our results showed that the increase in plant density decreased the grain-filling rate without a significant change in the active filling phase. Increasing N fertilizer input increased both the grain-filling rate and active filling phase. In particular, the increase in the maize grain-filling rate tended to be stronger with increased N input under high planting density (HD). The first grain-filling period (the lag grain-filling period) determines the potential kernel weight size (Hisse et al., 2019). Our study showed that in this period, the effect of increased N input on the kernel weight (i.e., the demand of the sink) significantly increased and advanced the completion of the lag grain-filling period, especially under HD conditions (Figure 4). The grain sink demand also adjusted the ^13^C-photosynthate transport ratio of vegetative organs and translocation from leaves to grains (Figure 5). Therefore, the effect of increased N fertilizer input under high planting density might occur through a change in the grain-filling rate and an increase in the potential to increase grain weight, not an extension of the active period proposed in a previous study (Poneleit and Egli, 1979). Increased N input significantly accelerated the activation of grain filling and the sink demand so that the kernel weight significantly increased during the initial grain-filling stages.

Additionally, previous studies pointed out that the grain-filling rate is chiefly restricted by insufficient photosynthate supply and sink capacity limitation (Fischer and Wilson, 1975; Evans, 1993; Gebbing and Schnyder, 1999). However, other studies have clarified that the source and sink were not the dominant limiting factors for the grain filling rate of maize under appropriate N input and without abiotic stresses, but the dominant limiting factors was the dry matter (DM) accumulation and how much of assimilation distribute to each organ (Pan et al., 2011; Zhu et al., 2011; Gasura et al., 2013; Shen et al., 2017). Additionally, previous study indicated that the assimilation source of grain filling mainly came from the current assimilation post-silking and the vegetative organ stored in the pre-silking (Fu et al., 2011; Tan et al., 2020). In this study, although translocation efficiency of vegetative organs at the at the silking (R1) stage to the milking (R3) stages was higher than that at the R3 to maturity (R6) stages, the grain translocation efficiency was only 2.3% (R1 to R3) to 17.2% (R3 to R6) during the whole grain-filling period of maize, and with increased N input, this efficiency decreased. Moreover, compared with N0, increased N input significantly enhanced the accumulation of postsilking DM (Figure 6) as well as the MTE (Figure 3), particularly under HD, increased 170.3% for the postsilking DM (R1 to R6) and increased 30.4% for the MTE. These result indication that the higher grain filling rate and grain yield may were attributed to the accumulation of DM and the MTE (i.e., current assimilation post-silking transport) rate rather than the vegetative organ stored in pre-silking, because of the low vegetative organ translocation efficiency cannot meet the assimilation required for grain filling rate and grain yield formation.

As important channels of matter transport in crops, vascular bundles can increase the accumulation of postsilking DM, adjusting the distribution of photosynthetic products in various organs (Shane et al., 2000; Noguchi et al., 2005). Previous studies showed that N application increased the number and area of small vascular bundles in the phloem in certain positions (panicle neck or cob), which was beneficial for matter transport and grain yield improvement (He et al., 2005; Terao et al., 2010). However, increased N input how to change the structure of the vascular bundle and the proportion of the phloem in the structure of the vascular bundle, and the relationship between the changed structure and the grain filling is still unclear. The results from this study suggest that the increase in N application significantly increased the number and area of small vascular bundles, which led to an increase in the proportion of the small vascular bundle area to the total vascular bundle area (PSVB), especially in peduncles and cobs (Table 1). Furthermore, N application coordinated the relationship between the proportion of and small vascular bundle areas to the total vascular bundle area and the MTE, which stimulated ^13^C-photosynthate allocation to grains to meet the sink (kernel) demand (Figure 5). Additionally, the accumulation of postsilking DM and grain-filling rate were improved by increasing N application, which could be explained by the change in the MTE. A positive relationship was found between MTE and vascular bundles in this study (Figure 7). These results suggest that PSVB in peduncles and cobs improved through an increase in the N supply, which was advantageous for MTE, contributing to the accumulation of DM, the grain-filling rate and the grain yield of maize under intensive planting.

## Conclusion

The increase in N application significantly improved the mean grain-filling rate, postsilking dry matter of maize and ^13^C-photosynthate allocation to grains at maturity and optimized the transport system of vascular bundles between organs as well, further coordinating the relationship between the big and small vascular bundles in peduncles and cobs, which greatly enhanced the MTE after maize silking (16.3–30.4%). These results suggest that increasing N input mainly optimized the vascular bundle structure of the ear system which improvement the accumulation of postsilking dry matter and MTE to enhance the mean grain filling and grain yield in maize under high planting density. These results might provide pivotal physiological evidence for how N management changes the vascular bundle transport system and clarifying the effect of the matter transport system on grain filling and grain yield under intensive planting conditions.

## Data Availability Statement

The raw data supporting the conclusions of this article will be made available by the authors, without undue reservation.

## Author Contributions

HR collected the samples, analyzed the samples, and wrote the manuscript. YJ and MZ made a contribution to writing and editing of the manuscript. HQ and CL made a contribution on to design of the work, analysis, and revised the manuscript. All authors read and approved the article.

## Conflict of Interest

The authors declare that the research was conducted in the absence of any commercial or financial relationships that could be construed as a potential conflict of interest.
