# Supplementary figures and images for: Nitrogen Supply Regulates Vascular Bundle Structure and Matter Transport Characteristics of Spring Maize Under High Plant Density
Source: Front Plant Sci. 2021 Jan 8;11:602739. doi: 10.3389/fpls.2020.602739 (PMC7820718; doi:10.3389/fpls.2020.602739)

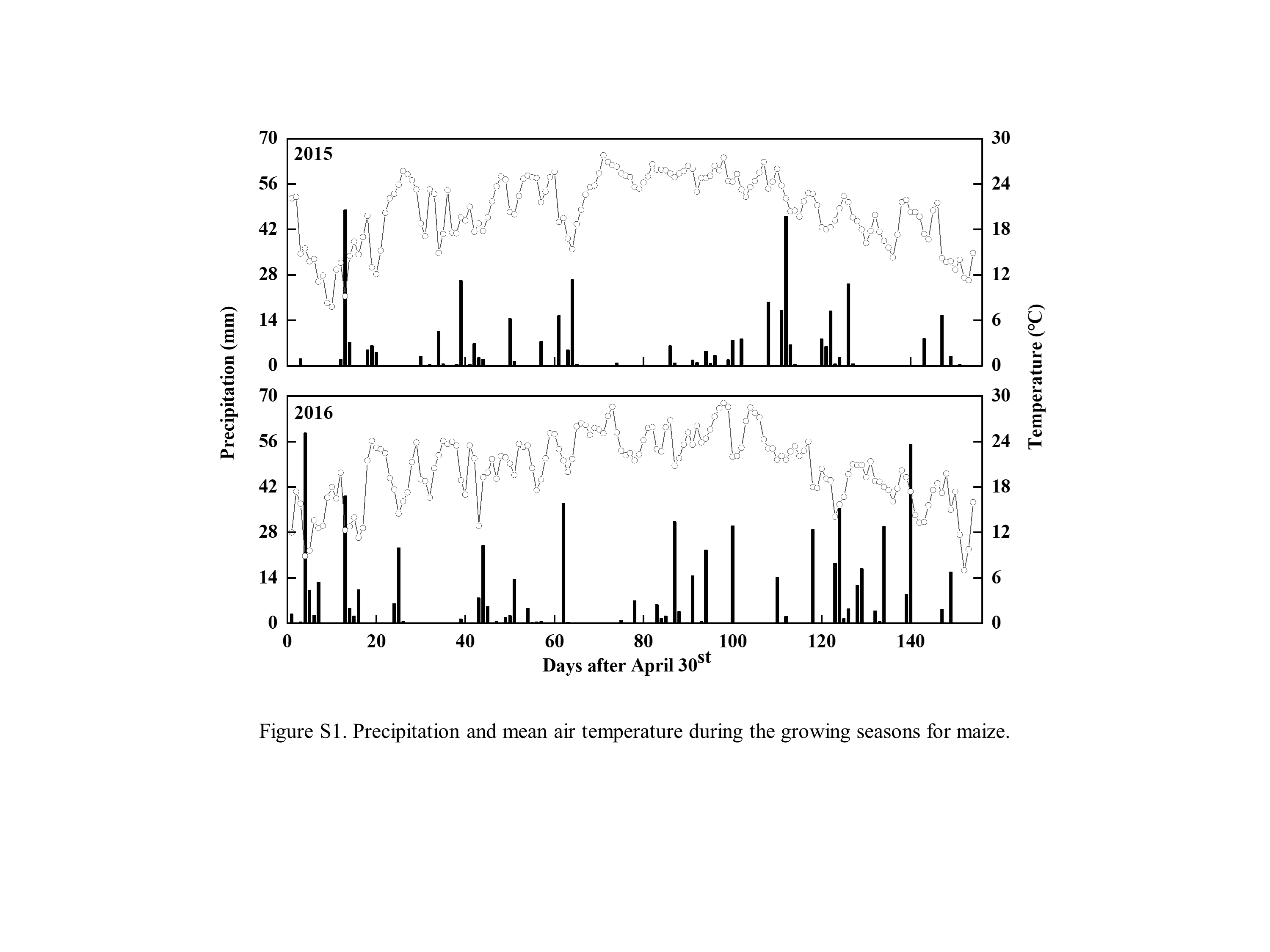

Supplement: Supplementary file 1 [file Image_1.TIF]
